# miR-155 and miR-146a collectively regulate meningitic *Escherichia coli* infection-mediated neuroinflammatory responses

**DOI:** 10.1186/s12974-021-02165-4

**Published:** 2021-05-13

**Authors:** Bo Yang, Ruicheng Yang, Bojie Xu, Jiyang Fu, Xinyi Qu, Liang Li, Menghong Dai, Chen Tan, Huanchun Chen, Xiangru Wang

**Affiliations:** 1 State Key Laboratory of Agricultural Microbiology, College of Veterinary Medicine, Huazhong Agricultural University, Wuhan, Hubei China; 2 Key Laboratory of Preventive Veterinary Medicine in Hubei Province, The Cooperative Innovation Center for Sustainable Pig Production, Wuhan, Hubei China; 3 Key Laboratory of Development of Veterinary Diagnostic Products, Ministry of Agriculture of the People’s Republic of China, Wuhan, Hubei China; 4 International Research Center for Animal Disease, Ministry of Science and Technology of the People’s Republic of China, Wuhan, Hubei China

**Keywords:** miR-155, miR-146a, *Escherichia coli*, Astrocytes, Neuroinflammation

## Abstract

**Background:**

*Escherichia coli* is the most common Gram-negative bacterium causing meningitis, and *E. coli* meningitis is associated with high mortality and morbidity throughout the world. Our previous study showed that *E. coli* can colonize the brain and cause neuroinflammation. Increasing evidence supports the involvement of miRNAs as key regulators of neuroinflammation. However, it is not clear whether these molecules participate in the regulation of meningitic *E. coli*-mediated neuroinflammation.

**Methods:**

The levels of miR-155 and miR-146a, as well as their precursors, in *E. coli*-infected astrocytes were measured using quantitative real-time PCR (qPCR). Overexpression and knockdown studies of miR-155 and miR-146a were performed to observe the effects on bacterial loads, cytokines, chemokines, and NF-κB signaling pathways. Bioinformatics methods were utilized to predict the target genes, and these target genes were validated using qPCR, Western blotting, and luciferase reporter system. In vivo knockdown of miR-155 and miR-146a was carried out to observe the effects on bacterial loads, inflammatory genes, astrocyte activation, microglia activation, and survival in a mouse model.

**Results:**

The levels of miR-155, miR-146a, and their precursors were significantly increased in astrocytes during *E. coli* infection*.* miR-155 and miR-146a were induced by the NF-κB-p65 signaling pathway upon infection. Overexpressing and inhibiting miR-155 and miR-146a in astrocytes did not affect the bacterial loads. Further, the in vitro overexpression of miR-155 and miR-146a suppressed the *E. coli*-induced inflammatory response, whereas the inhibition of miR-155 and miR-146a enhanced it. Mechanistically, miR-155 inhibited TAB2, and miR-146a targeted IRAK1 and TRAF6; therefore, they functioned collaboratively to modulate TLR-mediated NF-κB signaling. In addition, both miR-155 and miR-146a could regulate the EGFR–NF-κB signaling pathway. Finally, the in vivo suppression of *E. coli*-induced miR-155 and miR-146a further promoted the production of inflammatory cytokines, aggravated astrocyte and microglia activation, and decreased mouse survival time, without affecting the bacterial loads in the blood and brain.

**Conclusions:**

*E. coli* infection induced miR-155 and miR-146a, which collectively regulated bacteria-triggered neuroinflammatory responses through negative feedback regulation involving the TLR-mediated NF-κB and EGFR–NF-κB signaling pathways, thus protecting the central nervous system from further neuroinflammatory damage.

## Background

Bacterial meningitis remains an important cause of mortality and morbidity worldwide despite advances in antimicrobial chemotherapy and supportive care. The mortality and morbidity vary depending on the geographic location and patient age, and those living in low-income countries and newborns have a higher risk of mortality and morbidity [1–3]. *Escherichia coli* is the most common Gram-negative bacillary organism that causes meningitis, in particular during the neonatal period [4]. *E. coli* entry into the central nervous system (CNS) elicits the release of many factors from host cells including microglia, astrocytes, and infiltrating inflammatory cells, which exacerbates host cellular responses, leading to neuronal injury. Among all host cells, astrocytes are now emerging as those that can recruit, instruct, and retain leukocytes at sites of CNS insults [5]. Astrocytes can produce a variety of pro-inflammatory molecules such as cytokines, chemokines, growth factors, and small molecules. Our previous study showed that *E. coli* can colonize the brain and cause neuroinflammation [6]. A proper inflammatory response is beneficial to the host, whereas an excessive inflammatory response might result in damage. Therefore, the duration and intensity of the inflammatory response are vital to the host and should be maintained at an appropriate level.

MicroRNAs (miRNAs) play a vital role in posttranscriptional gene regulation by targeting 3’-untranslated regions (UTRs), which leads to translational tuning, repression, or degradation [7]. An increasing number of investigations has proved that miRNAs are important regulators of neuroinflammation. miR-155 and miR-146a are the most widely characterized miRNAs that modulate different stages of the innate immune response during inflammation and infection. Since each miRNA has multiple targets, a single miRNA could modulate a large number of proteins and thus can exert diverse effects in different situations. It has been widely reported that miR-155 contributes to pro-inflammatory signaling cascades by inhibiting SHIP-1 [8] and SOCS1 [9]; however, miR-155 also functions to deactivate the pro-inflammatory response by targeting IRF8 [10] and TAB2 [11]. miR-146a was initially reported to be upregulated in macrophages in response to the Toll-like receptor (TLR)-mediated NF-κB signaling pathway [12]. miR-146a is an important negative regulator of innate immune activation that functions by regulating TRAF6 and IRAK1 [12, 13]. However, given the redundant and highly cell-specific effects mediated by microRNA species, the precise functional implications of miR-155 and miR-146a expression during meningitic *E. coli* infection remain obscure.

In the current study, we demonstrated that the meningitic infection of astrocytes by *E. coli* could significantly upregulate miR-155 and miR-146a through NF-κB signaling, which in turn negatively regulated bacteria-triggered pro-inflammatory cytokine and chemokine production via TLR-mediated NF-κB signaling and the EGFR–NF-κB pathway. In vivo treatment of *E. coli*-infected mice with antagomir-155 or antagomir-146a augmented overall neuroinflammation and decreased survival time. These findings reveal that miR-155 and miR-146a are important inflammatory regulators of NF-κB activation during meningitic *E. coli* infections that function by inducing negative feedback with respect to TLR-mediated and EGFR-mediated immunity.

## Materials and methods

### Bacterial strain

The meningitic *E. coli* strain PCN033, which is a highly virulent cerebrospinal fluid isolate isolated in China in 2006 [14], was cultured aerobically in Luria-Bertani (LB) broth or on LB plates at 37 °C. This strain was shown to be able to cause host blood–brain barrier (BBB) disruption and severe neuroinflammation in vitro and in vivo [6].

### Cell culture and infection

The human astrocyte cell line U251 (a kind gift from Prof. Shengbo Cao in Huazhong Agricultural University, Wuhan, China) and HEK-293T cells (ATCC® CRL-3216™) were cultured in Dulbecco’s modified Eagle’s medium containing 10% heat-inactivated fetal bovine serum in a 37 °C incubator with a 5% CO_2_ atmosphere. U251 cells were cultured in 10-cm dishes until monolayer confluence. Confluent cells were washed three times with phosphate-buffered saline (PBS) and starved in serum-free medium for 16–18 h prior to infection. PCN033 overnight cultures were resuspended in serum-free medium and added to the starved U251 monolayer cells at a multiplicity of infection (MOI) of 10.

### Reagents

The anti-TAB2 antibody was purchased from Proteintech (Chicago, IL, USA). Anti-IRAK1 and anti-TRAF6 antibodies were from Sigma-Aldrich (St. Louis, MO, USA). Anti-β-actin antibody was from HuaAn Biotechnology Co., Ltd. (Hangzhou, China). Anti-NF-κB p65, anti-phospho-p65, HRP-conjugated anti-rabbit IgG, and anti-mouse IgG were obtained from Cell Signaling Technology (Danvers, MA, USA). The transfection reagent jetPRIME was purchased from Polyplus Transfection (Illkirch, France). has-miR-155 and has-miR-146a mimics (double-stranded RNA oligonucleotides), inhibitors (single-stranded chemically modified oligonucleotides), and control oligonucleotides were commercially synthesized by GenePharma (Suzhou, China). Cholesterol-conjugated and chemically modified mmu–miR-155 inhibitors (antagomir-155) and mmu–miR-146a inhibitors (antagomir-146a) were synthesized by GenePharma. The super electrochemiluminescence (ECL) kit was obtained from US Everbright Inc (Suzhou, China).

### Plasmid construction

The 3’ UTR sequences of target genes were amplified from U251 cDNA and cloned into the psiCheck-2 dual-luciferase reporter vector (Promega Madison, WI, USA). The promoter sequences of miR-155 and miR-146a were cloned into pGL3-basic (Promega Madison). The site-specific mutation plasmids were constructed by overlapping extension PCR. To construct the transcription factor expression vector, the coding regions of p65 were amplified from U251 cDNA and cloned into pCDNA3.1. All constructs were verified by sequencing.

### RNA interference

Small interfering RNAs (siRNAs) siRNA-TAB2, siRNA-IRAK1, siRNA-TRAF6, and the control siRNA were purchased from Gene Pharma. Transfection was performed with jetPRIME according to the manufacturer’s instructions. Cells were transfected with 50 nM of each siRNA.

### Transfection

U251 cells and HEK-293T cells were plated in six-well plates and grown to 70% confluence. Cells were subsequently transfected with plasmids and/or RNAs using jetPRIME according to the manufacturer’s instructions, followed by 24–48 h of incubation at 37 °C with 5% CO_2_.

### 3’ UTR luciferase reporter assays

HEK-293T cells were co-transfected with 200 ng psiCheck-2 target genes 3’ UTR luciferase reporter plasmid or psiCheck-2 mutant target genes 3’ UTR luciferase reporter plasmid, along with miRNA mimics or control (final concentration, 50 nM). After 24 h, cells were collected and assayed for both firefly and *Renilla* luciferase activities using the Dual-Luciferase Reporter System following the manufacturer’s instructions (Promega, Madison, WI, USA). The results were calculated as the ratio of *Renilla* luciferase activity to firefly luciferase activity and recorded as the mean + SD from three replicate wells.

### Promoter luciferase reporter assays

HEK-293T cells were co-transfected with 200 ng of full-length or mutant promoter firefly luciferase reporter constructs and 10 ng of *Renilla* luciferase vector (pRL-TK), along with the transcription factor expression vector or control. Luciferase activities were determined with the Dual-Luciferase Reporter Assay System according to the manufacturer’s protocol. The results were expressed as relative luciferase activity by normalizing firefly luciferase activity against *Renilla* luciferase activity and recorded as the mean + SD from three replicate wells.

### Animal infection and antagomir administration

The 28-day-old SPF KM mice were obtained from the experimental animal center at China Three Gorges University (Hubei Province, China). Mice were randomly assigned to four groups as follows: control (PBS); antagomir control-treated and *E. coli*-infected group (antagomir-ctrl + PCN033); antagomir-155-treated and *E. coli*-infected (antagomir-155 + PCN033); and antagomir-146a-treated and *E. coli*-infected (antagomir-146a + PCN033). For the antagomir, 60 mg/kg body weight of the antagomir was injected through the tail vein. After 24 h, mice were intravenously challenged with 1 × 10^7^ CFUs of *E. coli* strain PCN033 or an equal volume of PBS. At 6 h post-infection, mice were sacrificed and blood was collected for quantitative circulating bacterial cultures. Subsequently, mice were perfused, and brain samples were collected and processed for further assays. For the in vivo colonization assay, brains were weighed, homogenized, and plated to determine the bacterial counts.

### RNA extraction and quantitative real-time PCR (qPCR)

Total RNA was extracted using TRIzol® Reagent according to the manufacturer’s protocol. Following RNA extraction, 1 μg of RNA was reverse transcribed into cDNA using the HiScript II Q RT SuperMix (Vazyme, Nanjing, China). Real-time PCR was performed with the MonAmp™ SYBR Green qPCR Mix (RN04005M, Monad Biotech Co., Ltd, Wuhan, China) in accordance with the manufacturer’s instructions. The PCR conditions included an initial step at 50 °C for 2 min and 95 °C for 10 min, followed by 40 cycles of amplification and quantification (95 °C for 15 s, 60 °C for 1 min). The expression of all mRNA targets was normalized to *GAPDH* or *β-actin* levels, and the miRNA expression was normalized to *U6*. Primers used for qPCR in this study are listed in Table 1. The relative expression level was calculated by the 2^−ΔΔCT^ method, and the results are presented as the mean + SD. Each assay was performed independently three times in triplicate.

**Table 1:** Primers used for qPCR in this study

Gene	Forward (5’–3’)	Reverse (5’–3’)	Species
IRAK1	GCACCCACAACTTCTCGGAG	CACCGTGTTCCTCATCACCG	Human
TRAF6	TTGCCATGAAAAGATGCAGAGG	AGCCTGGGCCAACATTCTC	Human
TAB2	GCAGCAAAGGAACATCTAGCC	TGGACTGTTAAGTACAGGTGGA	Human
EGFR	TGCCACCTGTGCCATCCA	ACCACCAGCAGCAAGAGGAG	Human
GAPDH	TGCCTCCTGCACCACCAACT	CGCCTGCTTCACCACCTTC	Human
MIR155HG	GCGAGCAGAGAATCTACCT	TCTAAGCCTCACAACAACCT	Human
pri-mir146a	CTCCTCTGTCACCAAGTAA	CCTCTAACCTTCTGCCTAA	Human
IL1β	ATGATGGCTTATTACAGTGGCAA	GTCGGAGATTCGTAGCTGGA	Human
IL6	ACTCACCTCTTCAGAACGAATTG	CCATCTTTGGAAGGTTCAGGTTG	Human
TNF-α	CGAGTGACAAGCCTGTAG	GGACCTGGGAGTAGATGA	Human
MCP-1	CAGCCAGATGCAATCAATGCC	TGGAATCCTGAACCCACTTCT	Human
CXCL2	AGTGTGAAGGTGAAGTCC	CTTTCTGCCCATTCTTGAG	Human
IL1β	GCAACTGTTCCTGAACTCAACT	ATCTTTTGGGGTCCGTCAACT	Murine
IL6	TTCCATCCAGTTGCCTTCT	AAGCCTCCGACTTGTGAA	Murine
TNF-α	CCCTCACACTCAGATCATCTTCT	GCTACGACGTGGGCTACAG	Murine
MCP-1	TGTGAAGTTGACCCGTAA	TCCTACAGAAGTGCTTGAG	Murine
CXCL2	TGACTTCAAGAACATCCAGAG	CCTTGCCTTTGTTCAGTATCT	Murine
β-actin	GTCCCTCCTCTGATACCTTCCTC	CTGGCAGTGTCATTCACATCTTTCT	Murine

### Western blotting

Challenged U251 cells were lysed in RIPA buffer with a protease inhibitor cocktail (Sigma-Aldrich, USA), sonicated, and centrifuged at 12,000 rpm for 10 min at 4 °C. The protein concentration in the supernatant was measured using a BCA protein assay kit (Beyotime, China). Sodium dodecyl sulphate-polyacrylamide gel electrophoresis was performed, followed by protein transfer to polyvinylidene fluoride membranes using a Mini Trans-Blot Cell (Bio-Rad). Blots were probed with relevant antibodies, and the detection of proteins was conducted using ECL reagent. The densitometric analysis was performed using ImageJ software.

### Electrochemiluminescence (ECL) assays

Brain tissue samples from challenged mice were lysed in RIPA buffer (supplemented with protease inhibitor) and centrifuged at 12,000 rpm for 10 min to eliminate tissue debris. The supernatant was stored at – 80 °C and later used for the measurement of preselected cytokines and chemokines, including IL-1β, IL-6, TNF-α, MCP-1, and MIP-2 using the ECL V-Plex Proinflammatory Panel (mouse) kit (Meso Scale Discovery, Meso Scale Diagnostics, Rockville, MD, USA), following the manufacturer’s instructions.

### Immunofluorescence microscopy

U251 cells were transfected with miR-155, miR-146a, or miR-ctrl, and 24 h after transfection, cells were challenged with PCN033 at an MOI of 10 for 3 h, and cells were washed and fixed with 4% paraformaldehyde. The fixed cells were subsequently incubated with primary anti-p65 antibody and then with CY3-labeled goat anti-mouse IgG antibody. The plate was mounted and visualized using fluorescence microscopy.

Brain samples were collected, fixed in 4% formaldehyde solution for over 24 h, and embedded in paraffin. Sections were incubated overnight at 4 °C with primary antibodies against GFAP (glial fibrillary acidic protein) or IBA1 (ionized calcium-binding adaptor molecule 1). After washing, sections were incubated with appropriate secondary antibodies. Immunostainings were examined with a fluorescence microscopy. GFAP and IBA1 image analyses were performed using the ImageJ software (NIH, Bethesda, MD, USA).

### Histopathological examination

The brain samples were collected, fixed in 4% formaldehyde solution, and embedded in paraffin. Individual 6-μm sections were mounted on adhesive glass slides, dewaxed in xylene, and rehydrated in descending graded ethanol concentration for hematoxylin and eosin (H&E) staining according to the previous protocol [6].

### Statistical analysis

Data were expressed as the mean + SD unless otherwise specified. Statistical significance of the differences between each group was analyzed by Student’s *t* test or one-way analysis of variance (ANOVA) embedded in GraphPad Prism, version 7.0 (GraphPad Software Inc., La Jolla, CA, USA); *p* < 0.05 was considered statistically significant, *p* < 0.01 and *p* < 0.001 indicated extremely significant differences.

## Results

### *E. coli* infection upregulated the expression of miR-155 and miR-146a

**Fig. 1:**
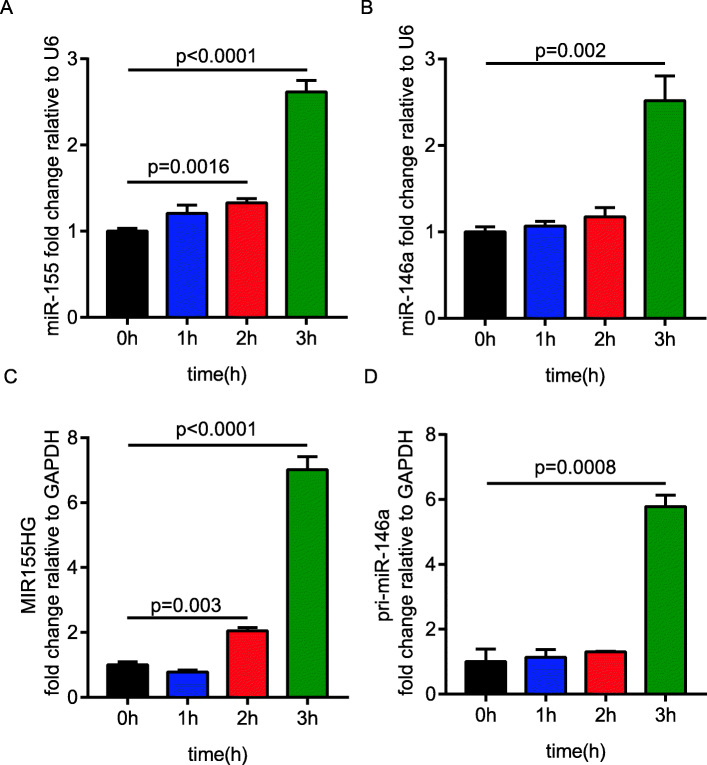
Meningitic *E. coli* PCN033 infection upregulates miR-155, miR-146a, and their precursors in astrocytes U251. **a** and **b** The transcriptional expression of miR-155 and miR-146a in U251 cells in response to PCN033 infection at a multiplicity of infection (MOI) of 10. U6 was used as the reference control. **c** and **d** The transcriptional expression of MIR155HG and pri-miR-146a in U251 cells in response to PCN033 infection at an MOI of 10. *GAPDH* was used as the reference control. Data represent the mean + standard deviation (SD) (*n* = 3/group). Statistical analysis was carried out by one-way ANOVA. *p* < 0.05 was considered statistically significant; *p* < 0.01 and *p* < 0.001 indicated extremely significant differences

Our previous miRNA transcriptional profiling of meningitic *E. coli*-infected astrocytes U251 showed that a number of miRNAs was differentially expressed upon *E. coli* infection. Among these miRNAs, miR-155 and miR-146a were found to be most significantly upregulated. Since miR-155 and miR-146a have been reported to be inflammation-associated molecules and *E. coli* infection can cause strong neuroinflammation, we therefore sought to explore their roles in meningitic *E. coli*-induced neuroinflammatory responses. First, we validated the expression of miR-155 and miR-146a in U251 cells stimulated by meningitic *E. coli* PCN033 for different times through real-time qPCR. The results revealed that miR-155 and miR-146a levels were significantly upregulated at 3 h, which was the later period of *E. coli* infection (Fig. 1a, b). Expression of the pri-miRNA MIR155HG and pri-mir-146a were in accordance with that of miR-155 and miR-146a (Fig. 1c, d).

### miR-155 and miR-146a were induced by the NF-κB signaling pathway

**Fig. 2:**
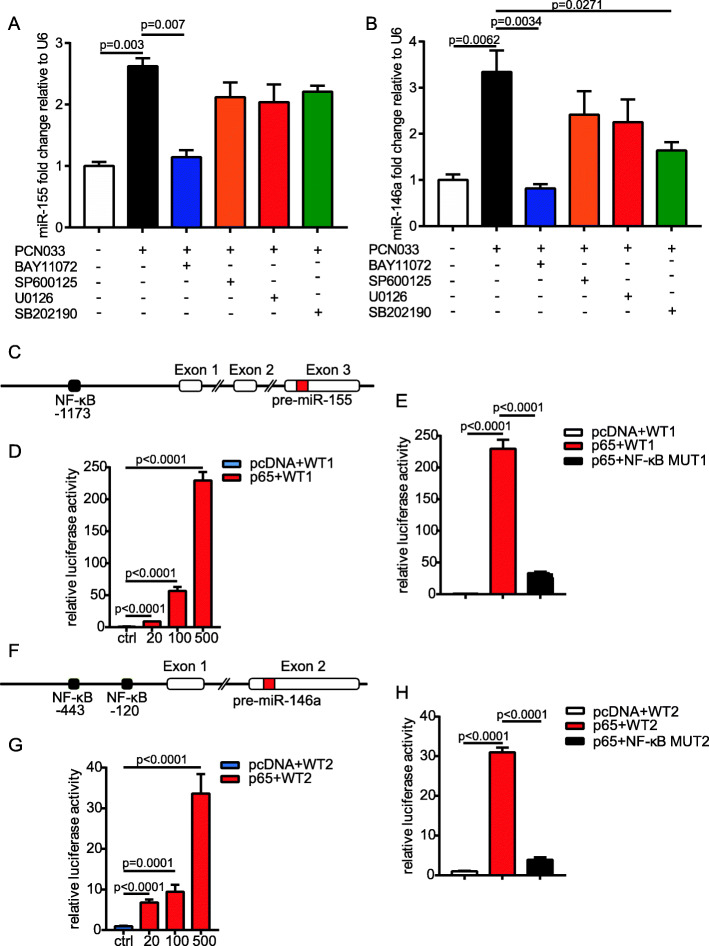
Analysis of signaling pathways and transcription factors involved in regulation of miR-155 and miR-146a expression. **a** and **b** U251 cells were infected with PCN033 or mock-infected in the presence of BAY11072, SP600125, U0126, and SB202190. The expression of miR-155 and miR-146a was detected by real-time qPCR. Data are presented as the mean + SD (*n* = 3/group). Statistical analysis was carried out by one-way ANOVA. **c** Schematic diagram of miR-155 genomic loci. A NF-κB binding site was predicted upstream of the MIR155HG transcription start site (+1). **d** Luciferase reporter assays were performed based on the co-transfection of a miR-155 promoter luciferase reporter (WT1), pRL-TK vector, and increasing concentrations of pcDNA3.1-p65 plasmid or empty vector control (pcDNA). Then, 24 h later, luciferase activity was measured, and the results were expressed as relative luciferase activity by normalizing firefly luciferase activity against *Renilla* luciferase activity, and results were recorded as the mean + SD from three replicate wells. **e** HEK-293T cells were co-transfected with a miR-155 promoter luciferase reporter or NF-κB mutation construct of the miR-155 promoter (NF-κB MUT1), along with pcDNA3.1-p65 plasmid. **f** Schematic diagram of miR-146a genomic loci. Two NF-κB binding sites were predicted upstream of the pri-miR-146a transcription start site (+1). **g** HEK-293T cells were co-transfected with a miR-146a promoter luciferase reporter (WT2), pRL-TK vector, and increasing concentrations of pcDNA3.1-p65 plasmid or empty vector control. **h** The miR-146a promoter luciferase reporter or NF-κB mutation construct of the miR-146a promoter (NF-κB MUT2), along with pcDNA3.1-p65 plasmid, were co-transfected into HEK-293T cells, and luciferase activity was measured. Error bars represented the SD calculated from the results of at least three independent experiments. Statistical analysis was carried out by one-way ANOVA. *p* < 0.05 was considered statistically significant; *p* < 0.01 and *p* < 0.001 indicated extremely significant differences

It has been widely reported that NF-κB and MAPK signaling pathways can regulate numerous important biological processes, including the biogenesis of miRNAs [15]. Considering that miR-155 and miR-146a are inflammation-associated miRNAs, we investigated whether these signaling pathways played roles in the regulation of miR-155 and miR-146a expression. We pretreated U251 cells with a set of specific inhibitors, including NF-κB (BAY-11072), JNK (SP600125), ERK1/2 (U0126), and p38 (SB202190), and then infected them with PCN033 for 3 h. As shown in Figs. 2a and b, the *E. coli* infection-induced upregulation of miR-155 and miR-146a was completely prevented by BAY-11072, but SP600125 and U0126 had no inhibitory effect on the expression of these miRNAs. SB202190 only had a limited inhibitory effect on the expression of miR-146a but had no effect on miR-155 levels. In addition, we noticed above that the pri-miRNA MIR155HG and pri-mir-146a exhibited the synchronous changes with the mature miR-155 and miR-146a (Fig. 1), suggesting that the upregulation of miR-155 and miR-146a might occur at the transcriptional level. To evaluate the transcriptional regulation of miR-155, we analyzed the promoter region of miR-155 to predict the potential transcription factor binding sites by using PROMO [16] (http://alggen.lsi.upc.es/cgi-bin/promo_v3/promo/promoinit.cgi?dirDB=TF_8.3). Combined with the results showing that miR-155 was modulated by NF-κB signaling pathways, we found an NF-κB binding site with high probability scores (Fig. 2c). As shown in Fig. 2d, the overexpression of p65 resulted in a concentration-dependent elevation of luciferase activity from the miR-155 promoter, but mutation of the NF-κB binding site significantly decreased miR-155 promoter activity (Fig. 2e). Similarly, the promoter region of miR-146a was analyzed and two NF-κB sites were found (Fig. 2f). It was observed from Figs. 2g and h that the overexpression of p65 enhanced the miR-146a promoter activity in a dose-dependent manner and the effect was abrogated by a mutation disturbing the two NF-κB element binding sites. Taken together, these results indicate that NF-κB signaling plays critical roles in the regulation of miR-155 and miR-146a by directly binding their promoters.

### miR-155 and miR-146a inhibited *E. coli*-induced pro-inflammatory factors production and NF-κB signaling

**Fig. 3:**
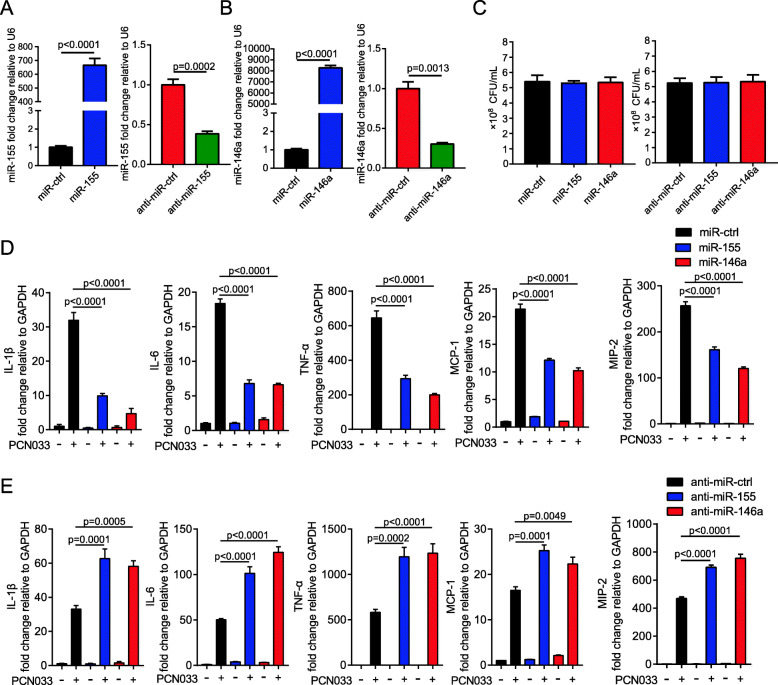
miR-155 and miR-146a repress meningitic *E. coli* PCN033-induced pro-inflammatory mediator expression. **a** and **b** U251 cells were transfected with mimic controls (miR-ctrls), miR-155 mimics (miR-155), miR-146a mimics (miR-146a), inhibitor controls (anti–miR-ctrl), miR-155 inhibitors (anti–miR-155), or miR-146a inhibitors (anti–miR-146a) for 24 h. The expression of miR-155 and miR-146a was detected by qPCR. U6 was used as an internal control. Data represented the mean + SD (*n* = 3/group). Statistical analysis was carried out by Student’s *t* test. **c** U251 cells were transfected as in **a** and **b** and infected with PCN033 at a MOI of 10 for 3 h. The survival bacteria were determined by plating on LB agar plates. **d** and **e** U251 cells were transfected as in **a** and **b** and infected with PCN033 at a MOI of 10 for 3 h. The expression of *IL-1β*, *IL-6*, *TNF-α*, *MCP-1*, and *MIP-2* were determined by qPCR. *GAPDH* was used as the internal reference. Data represented the mean + SD (*n* = 3/group). Statistical analysis was carried out by one-way ANOVA. *p* < 0.05 was considered statistically significant; *p* < 0.01 and *p* < 0.001 indicated extremely significant differences

**Fig. 4:**
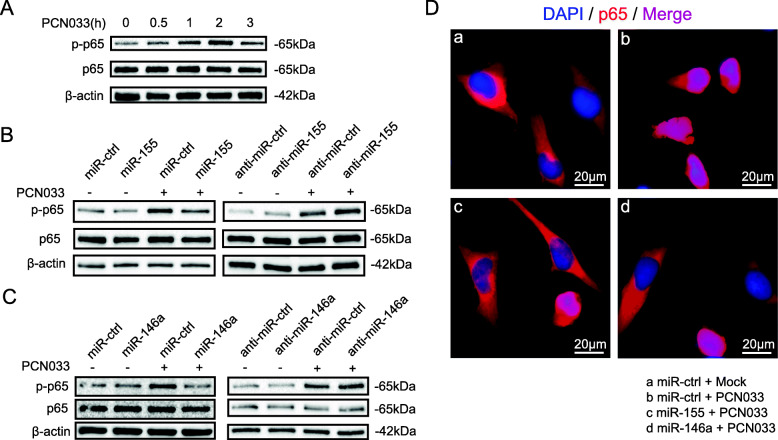
miR-155 and miR-146a suppress meningitic *E. coli* PCN033-induced activation of NF-κB signaling. a Western blot analysis of NF-κB p65 and phosphorylated p65 in U251 cells in response to PCN033 infection at different time points. β-actin was used as a loading control. b and c U251 cells were transfected as in Figs. 3a and b and infected with PCN033 at a MOI of 10 for 2 h; the protein levels of NF-κB p65 and phosphorylated p65 were determined by Western blotting (*n* = 3/group). d U251 cells were transfected with miR-ctrl, miR-155, or miR-146a and infected with PCN033. Translocation of the p65 subunit was detected by fluorescence microscopy. The p65 was labeled in red, and the cell nucleus was stained in blue with DAPI. Scale bar indicated 20 μm

To investigate the potential role of miR-155 and miR-146a in the response to *E. coli* infection, we assessed their effects on *E. coli*-induced inflammatory gene expression in astrocytes. Using the chemically synthesized mimics or inhibitors, we succeeded in up or downregulating the levels of miR-155 and miR-146a (Fig. 3a, b). We found that transfecting mimics and inhibitors of miR-155 and miR-146a had no influence on bacterial loads (Fig. 3c). Results showed that expression of the inflammatory cytokines IL-1β, IL-6, and TNF-α, as well as chemokines MCP-1 and MIP-2, were decreased after miR-155 or miR-146a mimics treatment (Fig. 3d). In contrast, the inhibitor of miR-155 or miR-146a increased these cytokines and chemokines (Fig. 3e). NF-κB-mediated pro-inflammatory gene expression plays an important role in the innate immune response against bacterial infection. *E. coli*-infected astrocytes exhibited the time-dependent upregulation of NF-κB p65 subunit phosphorylation, whereas p-p65 was decreased at 3 h, which was the timepoint that miR-155 and miR-146a were significantly increased (Fig. 4a). To determine whether miR-155 and miR-146a affect the NF-κB signaling pathway, we investigated their effects on phosphorylation of the p65 subunit in U251 cells. As shown in Figs. 4b and c, the infection-caused phosphorylation p65 in astrocytes transfected with miR-155 or miR-146a mimics was significantly decreased compared with that in cells transfected with miR-ctrl. Moreover, transfection of the miR-155 or miR-146a inhibitor promoted the phosphorylation p65. In addition, using immunofluorescence microscopy, we observed p65 translocation from the cytoplasm to the nucleus upon *E. coli* infection, which could be partly prevented by the transfection of miR-155 or miR-146a (Fig. 4d). These results showed that miR-155 and miR-146a play an inhibitory role in *E. coli*-induced pro-inflammatory factor production by negatively modulating NF-κB signaling.

### TAB2 was the functional target of miR-155

**Fig. 5:**
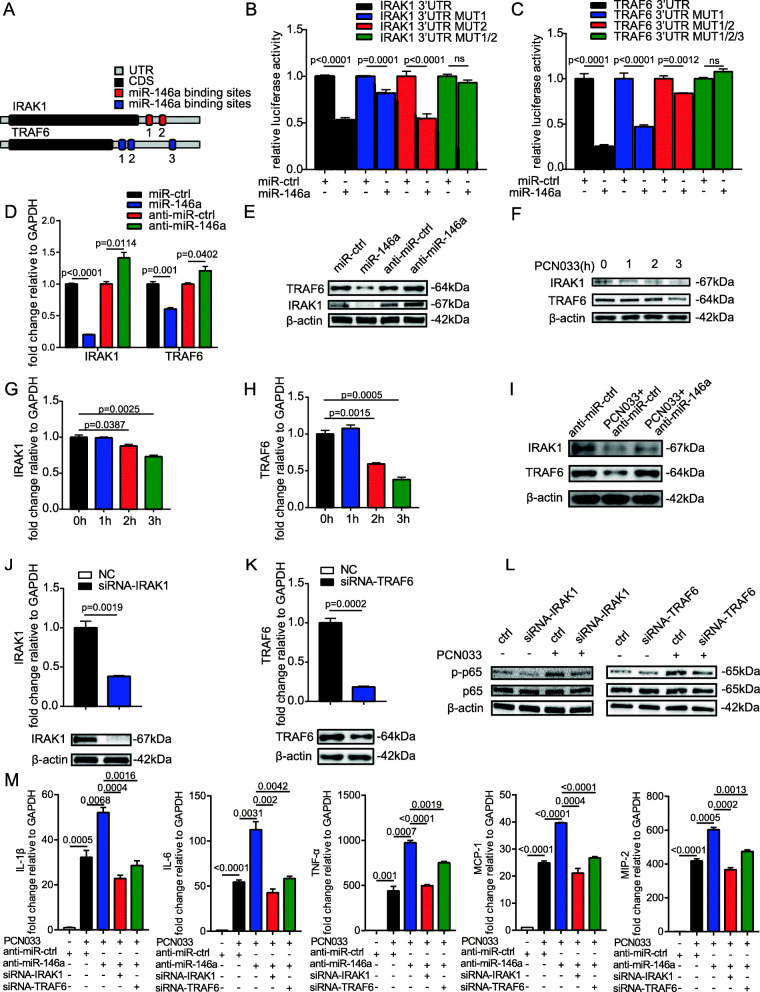
TAB2 is the functional target of miR-155. **a** Predicted miR-155 binding site in 3’ UTR of *TAB2*. Seven mutated nucleotides of the target site were indicated in red. **b** HEK-293T cells were co-transfected with miR-155 mimics or miR-ctrl, along with the wild-type or mutated *TAB2* 3’ UTR luciferase reporter plasmid, and assessed for luciferase activity at 24 h after transfection. Relative luciferase activity was expressed as the *Renilla* luciferase activity normalized to firefly luciferase activity. **c** and **d** The mRNA and protein levels of TAB2 were determined in U251 cells after 24 h of transfection with miR-155, anti-miR-155, or their corresponding control oligonucleotides. **e** and **f** The mRNA and protein levels of TAB2 were determined in PCN033-infected U251 cells. **g** U251 cells were transfected with anti-miR-155 or anti-miR-ctrl and then infected with PCN033 for 3 h. The expression of TAB2 was determined by Western blotting. **h** The mRNA and protein levels of TAB2 were determined in U251 cells after 24 h of transfection with siRNA-TAB2 or negative control (NC). **i** U251 cells were transfected with siRNA-TAB2 or NC and then infected with PCN033; the protein levels of NF-κB p65 and phosphorylated p65 were determined by Western blotting. **j** U251 cells were transfected with anti-miR-ctrl, anti-miR-155, or anti-miR-155, along with siRNA-TAB2, and then infected with PCN033 for 3 h. The expression of *IL-1β*, *IL-6*, *TNF-α*, *MCP-1*, and *MIP-2* was determined by qPCR. All data were represented as mean + SD (*n* = 3/group). Statistical analysis was carried out by Student’s *t* test or one-way ANOVA. *p* < 0.05 was considered statistically significant; *p* < 0.01 and *p* < 0.001 indicated extremely significant differences

Generally, miRNAs exert their regulatory functions by modulating translation of their target genes by binding the 3’ UTR of mRNA. Considering the results indicating that miR-155 can inhibit NF-κB activity, we first searched potential targets that are associated with inflammation. MiRNA target prediction software TargetScan showed that TAB2 had potential seed matches for miR-155. TAB2 is an adaptor molecule of the TLR/IL-1 signaling cascade that facilitates IL-1-dependent TNF receptor-associated factor 6 (TRAF6) ubiquitination and assembles the IL-1 signaling complex; thus, we further investigated the relationship between miR-155 and TAB2. A luciferase reporter carrying a putative binding site of the *TAB2* 3’ UTR along with a mutant construct with 7-bp mutations in the seed region were generated (Fig. 5a), and the results shown in Fig. 5b indicated that the transfection of miR-155 significantly inhibited the luciferase activity of the wild-type *TAB2* 3’ UTR construct but had no effect on the luciferase activity from the mutant construct. To further authenticate the negative correlation between miR-155 and TAB2, the expression of TAB2 was measured in U251 cells transfected with miR-155 mimics or inhibitors. As shown in Figs. 5c and d, the overexpression of miR-155 significantly suppressed both mRNA and protein levels of TAB2, whereas the miR-155 inhibitor led to upregulated TAB2 mRNA and protein levels. In addition, the mRNA and protein expression of TAB2 were significantly downregulated in U251 cells upon *E. coli* infection (Fig. 5e, f), which was the opposite trend compared with that of miR-155. Moreover, we measured the protein level of TAB2 in U251 cells transfected with anti-miR-155 after *E. coli* infection. As shown in Fig. 5g, the *E. coli* infection-induced downregulation of TAB2 was restored by inhibiting miR-155, demonstrating that miR-155 can regulate TAB2 during *E. coli* infection. To further confirm that miR-155 exerts its effect through TAB2, we subsequently knocked down TAB2 in U251 cells using siRNA. As seen in Fig. 5h, TAB2 was successfully inhibited. As expected, the transfection of TAB2 siRNA resulted in decreased phosphorylation of NF-κB p65 upon *E. coli* infection compared with that with the transfection of siRNA control (Fig. 5i). Importantly, the augmented expression of inflammatory genes (*IL-1β*, *IL-6*, *TNF-α*, *MCP-1*, and *MIP-2*) induced by the inhibition of miR-155 was decreased by suppressing TAB2 (Fig. 5j). Collectively, these results demonstrate that TAB2 is the functional target of miR-155.

### IRAK1 and TRAF6 were functional targets of miR-146a

**Fig. 6:**
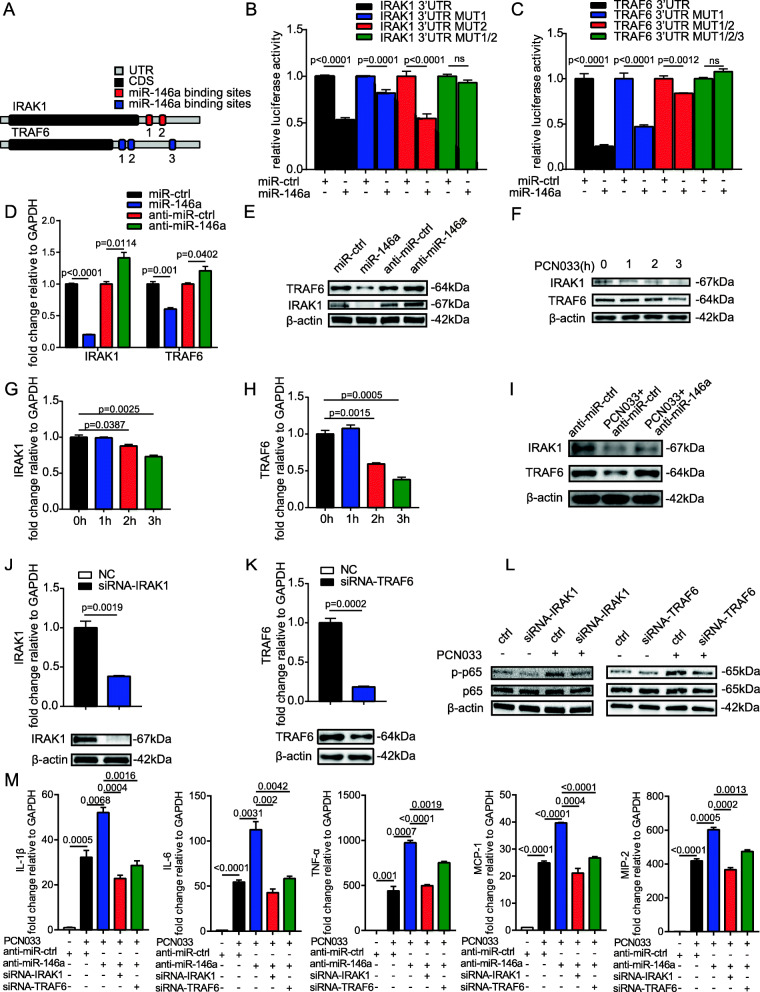
IRAK1 and TRAF6 are functional targets of miR-146a. **a** Predicted miR-146a binding sites in 3’ UTRs of *IRAK1* and *TRAF6*. *CDS*, coding sequence. **b** and c HEK-293T cells were co-transfected with miR-146a mimics or miR-ctrl, along with the wild-type or mutated *IRAK1* 3’ UTR or *TRAF6* 3’ UTR luciferase reporter plasmids, and assessed for luciferase activity 24 h after transfection. **d** and **e** The mRNA and protein levels of IRAK1 and TRAF6 were determined in U251 cells after 24 h of transfection with miR-146a, anti-miR-146a, or their corresponding control oligonucleotides. **f**–**h** The mRNA and protein levels of IRAK1 and TRAF6 were determined in PCN033-infected U251 cells. **i** U251 cells were transfected with anti-miR-146a or anti-miR-ctrl and then infected with PCN033 for 3 **h**. The expression of IRAK1 and TRAF6 was determined by Western blotting. **j** and **k** The mRNA and protein levels of IRAK1 and TRAF6 were determined in U251 cells after 24 h of transfection with siRNA-IRAK1, siRNA-TRAF6, or negative control (NC). **l** U251 cells were transfected with siRNA-IRAK1, siRNA-TRAF6, or NC and then infected with PCN033; the protein levels of NF-κB p65 and phosphorylated p65 were determined by Western blotting. **m** U251 cells were transfected with anti-miR-ctrl, anti-miR-146a, or anti-miR-146a, along with siRNA-IRAK1, or anti-miR-146a, along with siRNA-TRAF6, and then infected with PCN033 for 3 h. The expression of *IL-1β*, *IL-6*, *TNF-α*, *MCP-1*, and *MIP-2* was determined by qPCR. All data were represented as mean + SD (*n* = 3/group). Statistical analysis was carried out by Student’s *t* test or one-way ANOVA. *p* < 0.05 was considered statistically significant; *p* < 0.01 and *p* < 0.001 indicated extremely significant differences

IRAK1 and TRAF6 have been reported to be important targets of miR-146a in monocytes and macrophages during the innate immune response [12, 17]. Since miR-146a was experimentally proven to be associated with meningitic *E. coli* infection, whether it also targeted IRAK1 and TRAF6 in astrocytes needed further investigation. Bioinformatic analysis showed that there are two binding sites in the IRAK1 3’ UTR and three binding sites in the *TRAF6* 3’ UTR (Fig. 6a). We found a significant decrease in luciferase activity in HEK-293T cells co-transfected with miR-146a mimics and the wild-type *IRAK1* 3’ UTR luciferase reporter plasmid, and miR-146a mimics still significantly downregulated the relative luciferase activity from the IRAK1-3’ UTR-MUT2 construct but had mild effects on the IRAK1-3’ UTR-MUT1 construct (Fig. 6b). This result showed that binding site 1 in the 3’ UTR of *IRAK1* is a vital targeting site of miR-146a. Similarly, we constructed a wild-type *TRAF6* 3’ UTR luciferase reporter plasmid with a single site mutation, a double site mutation, and a triple site mutation. As shown in Fig. 6c, the overexpression of miR-146a markedly inhibited luciferase activity from the wild-type *TRAF6* 3’ UTR luciferase reporter plasmid, whereas the suppression of luciferase activity was restored gradually in the presence of site mutations, revealing that all three binding sites play important roles in combination with miR-146a. In addition, overexpressing miR-146a significantly suppressed IRAK1 and TRAF6 expression in U251 cells, both at the mRNA and protein levels, whereas miR-146a inhibition led to the increased expression of IRAK1 and TRAF6 (Fig. 6d, e). We also measured the expression of IRAK1 and TRAF6 along with the *E. coli* infection and found that they were decreased in U251 cells upon infection (Fig. 6f–h). Moreover, miR-146a was determined to regulate the expression of IRAK1 and TRAF6 during *E. coli* infection (Fig. 6i). To further verify the function of miR-146a targets, we subsequently knocked down the expression of IRAK1 and TRAF6 at the mRNA and the protein levels (Fig. 6j, k). As shown in Fig. 6l, the inhibition of both IRAK1 and TRAF6 reduced the phosphorylation of p65 induced by PCN033, as compared with that with their controls. Furthermore, inhibiting miR-146a promoted the expression of PCN033-induced inflammatory genes, including *IL-1β*, *IL-6*, *TNF-α*, *MCP-1*, and *MIP-2*, and these genes were notably reduced by interfering with IRAK1 or TRAF6 (Fig. 6m). Taken together, miR-146a exerts an inhibitory effect on inflammation by targeting IRAK1 and TRAF6. miR-155 and miR-146a targeted different molecules in the signaling cascade that downstream of TLR, including TAB2, IRAK1, and TRAF6, thereby collectively modulating the TLR-mediated inflammatory response.

### miR-155 and miR-146a collectively modulated EGFR–NF-κB signaling

**Fig. 7:**
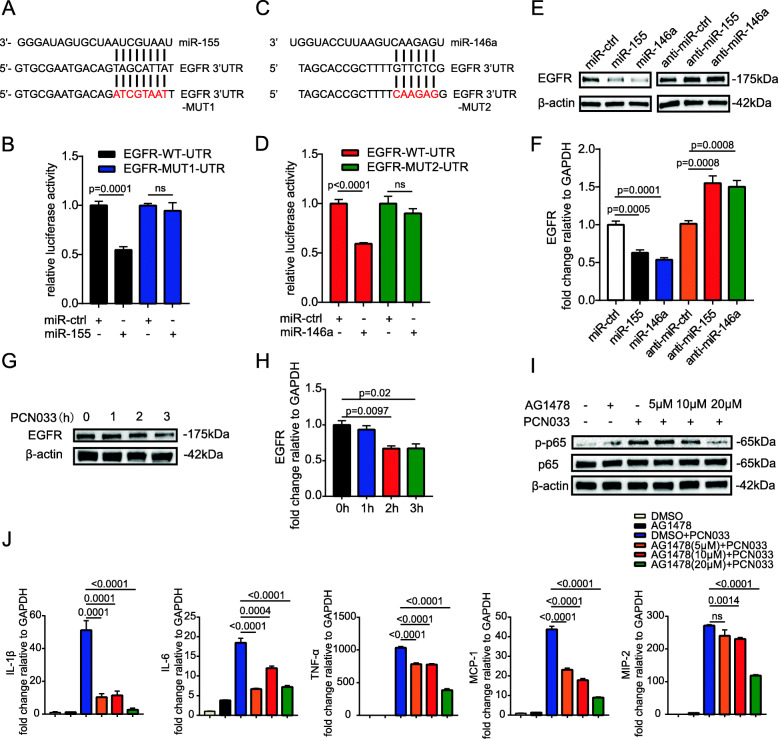
miR-155 and miR-146a collectively modulate EGFR–NF-κB signaling. **a** Predicted miR-155 binding site in 3’ UTR of *EGFR*. Eight mutated nucleotides of the target site were indicated in red. **b** HEK-293T cells were co-transfected with miR-155 mimics or miR-ctrl, along with the wild-type or mutated *EGFR* 3’ UTR luciferase reporter plasmid (EGFR 3’ UTR-MUT1). **c** Predicted miR-146a binding site in 3’ UTR of *EGFR*. Six mutated nucleotides of the target site are indicated in red. **d** HEK-293T cells were co-transfected with miR-146a mimics or miR-ctrl, along with the wild-type or mutated *EGFR* 3’ UTR luciferase reporter plasmid (EGFR 3’ UTR-MUT2). **e** and **f** The mRNA and protein levels of EGFR were determined in U251 cells after 24 h of transfection with miR-155, miR-146a, anti-miR-155, anti-miR-155, or their corresponding control oligonucleotides. **g** and **h** The mRNA and protein levels of EGFR were determined in PCN033-infected U251 cells. **i** and **j** U251 cells were infected with PCN033 or mock-infected in the presence of increasing concentrations of the EGFR inhibitor AG1478, and the protein levels of NF-κB p65 and phosphorylated p65 were determined by Western blotting, while the mRNA levels of *IL-1β*, *IL-6*, *TNF-α*, *MCP-1*, and *MIP-2* were determined by qPCR. All data were represented as mean + SD (*n* = 3/group). Statistical analysis was carried out by Student’s *t* test or one-way ANOVA. *p* < 0.05 was considered statistically significant; *p* < 0.01 and *p* < 0.001 indicated extremely significant differences

When we used bioinformatics tools to predict the targets of miR-155 and miR-146a, interestingly, we found that EGFR possibly combines with both miR-155 and miR-146a. Moreover, our previous studies showed that EGFR contributes to bacterial-induced neuroinflammation by triggering the MAPK-ERK1/2 and NF-κB signaling pathways [18]. Thus, we investigated the regulative relationship between EGFR and miRNAs. Bioinformatics prediction showed that miR-155 and miR-146a contained respective binding sites for EFGR (Fig. 7a, c), and the overexpression of miR-155 or miR-146a inhibited luciferase expression from the wild-type *EGFR* 3’ UTR. In contrast, the activity of luciferase constructs containing the mutant 3’ UTR of *EGFR* was not inhibited by overexpressing miR-155 or miR-146a (Fig. 7b, d). In addition, miR-155 and miR-146a significantly decreased mRNA and protein levels of EGFR, whereas the inhibition of miR-155 or miR-146a promoted the expression of EGFR (Fig. 7e, f). Besides, U251 cells exhibited the significant downregulation of EGFR in response to meningitic *E. coli* infection (Fig. 7g, h). All these results suggested that EGFR is a potential target of miR-155 and miR-146a. To further investigate whether EGFR participates in the inflammatory process in response to *E. coli* infection, we pretreated U251 cells with 5, 10, or 20 μM of the EGFR inhibitor AG1478 or DMSO as a control, and explored whether NF-κB signaling pathways were involved in the EGFR-mediated inflammatory response. Results showed that the p65 phosphorylation induced by *E. coli* infection was notably attenuated by AG1478 in a dose-dependent manner (Fig. 7i), and correspondingly, as shown in Fig. 7 j, *E. coli*-induced IL-1β, IL-6, TNF-α, MCP-1, and MIP-2 expression was significantly suppressed by treatment with AG1478 in a dose-dependent manner. Taken together, these results indicated that EGFR is a target of miR-155 and miR-146a and that blocking EGFR activity could markedly alleviate downstream NF-κB signaling pathways, thus suppressing the production of pro-inflammatory cytokines and chemokines.

### Antagomir-155 and antagomir-146a aggravated inflammatory responses and reduced survival duration in *E. coli*-infected mice

**Fig. 8:**
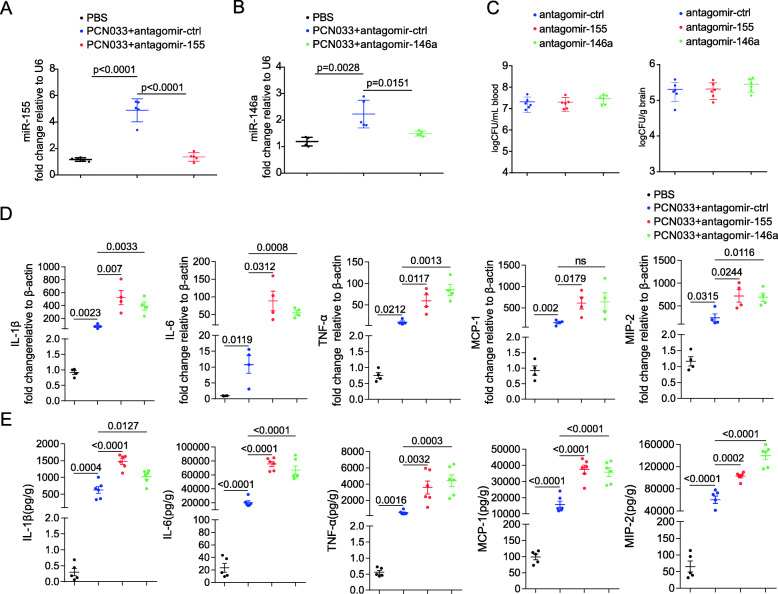
Antagomir-155 and antagomir-146a promote the production of inflammatory cytokines and chemokines in the *E. coli* infected mice. Mice were injected with antagomir-155, antagomir-146a, or antagomir-ctrl 24 h before PCN033 challenge. At 6 h post-challenge, the brain tissues were collected. **a** and **b** qPCR analysis of the expression of miR-155 and miR-146a from brain tissues (*n* = 5–6/group). Data represented the mean ± SD. Statistical analysis was carried out by one-way ANOVA. **c** Bacterial loads in the blood and brain from three groups of mice were compared. **d** qPCR analysis of *IL-1β*, *IL-6*, *TNF-α*, *MCP-1*, and *MIP-2* in brain tissues. **e** ECL analysis of IL-1β, IL-6, TNF-α, MCP-1, and MIP-2 in brain tissues. Data represented the mean ± SD. Statistical analysis was carried out by one-way ANOVA. *p* < 0.05 was considered statistically significant; *p* < 0.01 and *p* < 0.001 indicated extremely significant differences

**Fig. 9:**
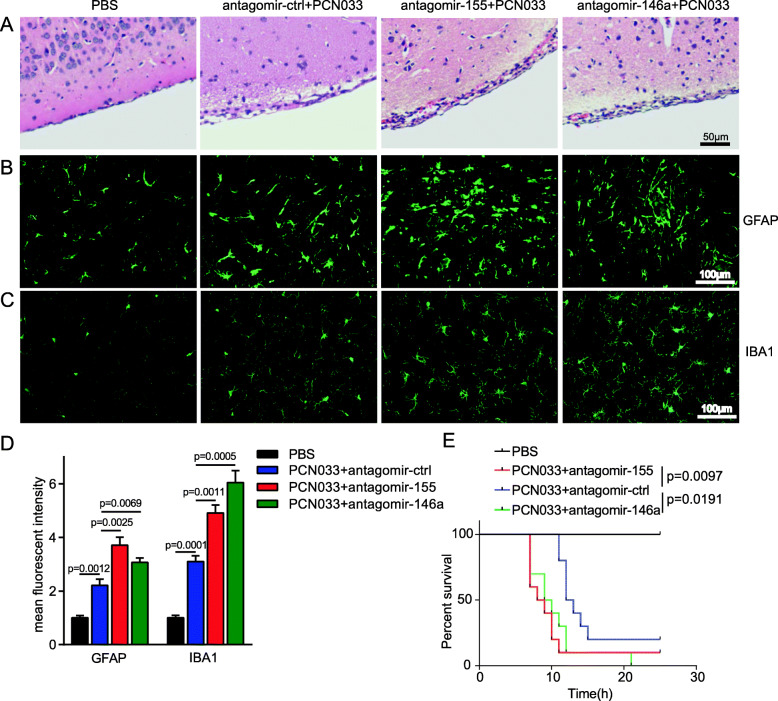
Antagomir-155 and antagomir-146a aggravate neuroinflammation and reduce survival duration in the *E. coli*-infected mice. **a** H&E staining of brain sections. The scale bar indicated 50 μm. **b** and **c** Immunofluorescence analysis of GFAP and IBA1 in brain sections; the scale bar indicated 100 μm. **d**. Quantification of the mean fluorescence intensity of GFAP- and IBA1-positive cells in the brain. Data represented the mean + SD. Statistical analysis was carried out by one-way ANOVA. **e** Survival of mice in each group was monitored for 24 h after tail vein injection of PCN033. Data were collected and shown as Kaplan–Meier survival curves (*n* = 10). Statistical analysis was carried out by Log–rank (Mantel-Cox) test. *p* < 0.05 was considered statistically significant; *p* < 0.01 and *p* < 0.001 indicated extremely significant differences

We further determined the effects of miR-155 and miR-146a in *E. coli*-infected mice using chemically modified antisense oligonucleotides specific for miR-155 (antagomir-155) and miR-146a (antagomir-146a), which can cross the BBB into the CNS via i.v. routes [19–21]. Antagomir-155 and antagomir-146a were delivered to mice via the tail vein 24 h prior to PCN033 infection, and mice showed no signs of discomfort. At 6 h post-challenge, mice exhibited typical neurological signs including trembling, circling, paddling, and opisthotonos [6], at which time mice were sacrificed and brain tissues were collected. As shown in Figs. 8a and b, the injection of antagomir-155 significantly downregulated the expression of miR-155 induced by *E. coli* infection in mouse brains, and antagomir-146a also suppressed the level of miR-146a. Decreasing the expression of miR-155 and miR-146a did not affect the bacterial loads in blood and brains of challenged mice (Fig. 8c). Compared with levels in mice injected with antagomir-ctrl, inflammatory cytokines, and chemokines including IL-1β, IL-6, TNF-α, MCP-1, and MIP-2 were further enhanced at the transcription and protein levels by antagomir-155 and antagomir-146a treatment (Fig. 8d, e). These results indicated that miR-155 and miR-146a altered the inflammatory responses in respond to *E. coli* infection, which is not relevant to bacterial growth or survival. In addition, H&E staining results showed that *E. coli* infection could induce meningeal thickening and neutrophil infiltration in the meninges, and these pathological phenomena were further exacerbated by treatment with antagomir-155 and antagomir-146a (Fig. 9a). Moreover, *E. coli* infection induced astrocytosis and microgliosis in mice, and these pathological phenomena were further exacerbated by antagomir-155 and antagomir-146a treatment (Fig. 9b–d). Based on these observations, we evaluated the effects of mir-155 and mir-146a on mouse lethality. As shown in Fig. 9e, the administration of antagomir-155 and antagomir-146a caused no significant changes in mouse mortality; however, the survival time was significantly decreased. In conclusion, inhibiting miR-155 and miR-146a in mice can further aggravate pro-inflammatory responses and lead to the reduced survival duration.

## Discussion

Astrocytes are the most abundant glial cells within the brain and are essential for brain homeostasis and neuronal functions [22, 23]. Astrocytes play vital roles in regulating innate and adaptive immune responses in the injured CNS. Our previous study showed that the meningitis-associated *E. coli* strain PCN033 could infect astrocytes U251, induce rapid inflammatory responses, and promote the expression of many pro-inflammatory mediators such as IL-1β, IL-6, IL-8, TNF-α, MCP-1, and MIP-2. We hypothesized that astrocytes would trigger inflammatory responses to recruit circulating immune cells to the sites of insults, thereby mediating immune elimination of the pathogen. However, exaggerated or persistent long-term inflammatory responses lead to pathological neuroinflammation, and thus it is essential to tightly regulate the inflammatory response to avoid further damage of the CNS.

Increasing research has revealed that miRNAs are essential regulators of various biological processes, including inflammation [24, 25]. Our previous comprehensive miRNA sequencing data showed that a group of miRNAs is differentially expressed upon PCN033 infection. Among these miRNAs, miR-155 and miR-146a exhibited the highest expression and were found to be the most significantly upregulated. Given that the regulatory role of miR-155 and miR-146a in the context of meningitic *E. coli* infection remained unknown, we selected these two molecules as targets for further experimentation. In this study, we demonstrated that miR-155 and miR-146a were highly upregulated by *E. coli* through NF-κB signaling and that miR-155 and miR-146a negatively regulated bacteria-induced pro-inflammatory responses. Importantly, we found that miR-155 combined with miR-146a to exert anti-inflammatory effects by targeting different key proteins in TLR signaling pathways and collectively regulate EGFR–NF-κB signaling.

TLRs play important roles in recognizing pathogens and initiating inflammatory responses during infection [26]. However, it is crucial to tightly modulate the TLR signaling pathways to avoid excessive inflammation. During this process, miRNAs can modulate the TLR signaling pathways by inhibiting key intracellular signaling proteins. A previous study and our work showed that miR-146a can inhibit IRAK1 and TRAF6. In addition, IRAK2, a kinase that is necessary for the persistence of NF-κB activation, has also been shown to be regulated by miR-146a [17]. In addition to miR-146a, miR-155 can target vital signaling proteins in TLR signaling pathways. MyD88 has been identified as a target of miR-155 in a model of *Helicobacter pylori* infection [27]. We also proved that TAB2 was an important target of miR-155. Moreover, Schulte et al. has verified that IKKƐ and NIK were targeted by miR-155 [28]. Consequently, miR-155 and miR-146a can work together to target different components of several TLR signaling pathways to avoid excessive pro-inflammatory responses. Besides, many miRNAs can also target signaling molecules in TLR pathways. For example, miR-302b suppressed bacteria-induced inflammatory responses by regulating IRAK4 [29]. The activity of NF-κB was tightly regulated by inhibitor of NF-κB kinases (IKKs), whereas IKKα was targeted by miR-223, and IKKβ was regulated by miR-199 [30, 31]. Further, many other miRNAs participated in the regulation of TLR signaling pathways through different mechanisms. Some miRNAs can directly modulate the expression of receptor expression. For example, let-7 miRNA family members let-7e and let-7i can regulate the expression of TLR4 [32, 33], and TLR3 and TLR4 expressions were regulated by miR-223 in granulocytes [34]. Some miRNAs can also target transcription factors to affect TLR-induced gene expression. For example, miR-155 targeted CEBPB to decrease G-CSF and IL-6 expression in splenocytes. Altogether, miRNAs could play crucial roles in controlling inflammation by inhibiting key proteins in the TLR signaling pathways.

EGFR is a member of the ErbB family, which is composed of four tyrosine kinase receptors, EGFR (ErbB1) and ErbB2–4 [35]. These four receptors are essential for modulating many biological processes, including cell survival, proliferation, and differentiation in many tissue types [36]. Despite the fact that EGFR has been mainly studied in the field of cancer, increasing studies have discovered diverse roles in pathogenic bacterial infections, such as regulating bacterial invasion, inflammation, and apoptosis [37–39]. Our recent research showed that the bacteria-induced transactivation of EGFR activated downstream signaling pathways NF-κB and MAPK-ERK1/2 in hBMECs, which subsequently initiated and mediated the inflammatory response. In this study, we blocked the function of EGFR by introducing the EGFR inhibitor AG1478 in astrocytes, resulting in the reduced expression of pro-inflammatory factors. This result suggests that EGFR also functions as an initiator of inflammatory responses in astrocytes. We further confirmed that NF-κB signaling pathways were influenced by EGFR activity. Importantly, we proved that EGFR was a common target of miR-155 and miR-146a, and thus, both can serve as anti-inflammatory miRNAs by targeting EGFR, subsequently inhibiting downstream NF-κB signaling pathways and eventually suppressing inflammatory cytokines and chemokines. EGFR has been reported as a target of miR-146a in cancer, and miR-146a can block pancreatic cancer cell invasion and metastasis by inhibiting EGFR and IRAK1 [40]. To our knowledge, this is the first study to show that miR-146a and EGFR are implicated in bacterial infection and the related immune response and that EGFR is also a target of miR-155.

Our in vivo data showed that inhibiting miR-155 and miR-146a in mice promoted the production of inflammatory cytokines, aggravated astrocyte and microglia activation, and decreased mouse survival time. However, the bacterial loads in the blood and brain remained unchanged. We propose that miR-155 and miR-146a can modulate the inflammatory responses of mice to respond to *E. coli* infection, rather than exerting direct antibacterial activity. In this study, almost all *E. coli*-challenged mice showed neurologic symptoms including trembling, circling, paddling, and opisthotonos. Besides, we observed the meningeal thickening and neutrophil infiltration in the meninges in response to the infection. Moreover, the production of pro-inflammatory cytokines and chemokines and the activation of astrocytes and microglia further supported the occurance of meningitis after hematogenous *E. coli* infection. Considering the important immunomodulation effects of miR-155 and miR-146a on *E. coli*-induced meningitis, miR-155 and miR-146a may be attractive candidates of new therapeutic interventions in the treatment of bacterial meningitis.

We found that miR-155 and miR-146a are simultaneously upregulated in astrocytes during the later period of *E. coli* infection. Therefore, a one-tier model was established in that NF-κB-regulated miR-155 and miR-146a collectively act via feedback to modulate TLR signaling and EGFR-NF-κB signaling. In contrast to our results, Schulte [28] identified a two-tier mechanism in LPS-stimulated macrophages; specifically, miR-146 was found to be activated at sub-inflammatory levels, whereas miR-155 was gradually induced to full expression at pro-inflammatory levels. Therefore, miR-155 acts as a final limit to the inflammatory response once the miR-146-dependent barrier to LPS-induced inflammation has been breached. Interestingly, a recent study showed that miR-155 and miR-146a can form a combined positive and negative regulatory loop regulating NF-κB activity [41]. Inflammatory stimuli lead to the activation of NF-κB, which rapidly activates miR-155. miR-155 acts as a positive regulator of NF-κB activity by inhibiting SHIP1 and SOCS1. As the inflammatory response develops, miR-146a levels accumulate to negatively regulate NF-κB activity, resulting in the attenuation of inflammatory gene and miR-155 expression. In addition, another study on rheumatoid arthritis (RA) showed that miR-155 and miR-146a were downregulated in Tregs from RA patients, and the decrease in miR-146a-induced pro-inflammatory effects prevailed over the counteracting effect of reduced miR-155 expression, resulting in a pro-inflammatory phenotype for Tregs in RA [42]. It seems that miR-155 and miR-146a play different roles in controlling inflammatory responses in different disease models, depending on the diversity of cell types and experimental conditions. This also sheds light on the importance of understanding the interactions between them and the contribution of miR-155 and miR-146a to specific inflammatory disorders.

## Conclusions

In this study, we demonstrated the roles of miR-155 and miR-146a in the progress of meningitic *E. coli* infection. miR-155 and miR-146a were found to collectively regulate bacteria-triggered neuroinflammatory responses via negative feedback regulation of the TLR-mediated NF-κB and EGFR–NF-κB signaling pathways. Our findings might suggest potential therapeutic targets to combat bacterial infection in future.

## Data Availability

There is no data, software, databases, and application/tool available apart from the reported in the present study. All data are provided in manuscript.

## Abbreviations

BBB: Blood–brain barrier
CNS: Central nervous system
*E. coli*: *Escherichia coli*
ECL: Electrochemiluminescence
ELISA: Enzyme-linked immunosorbent assay
FBS: Fetal bovine serum
IF: Immunofluorescence
IKK: Inhibitor of NF-κB kinase
LB: Luria-Bertani
miRNA: MicroRNA
PBS: Phosphate-buffered saline
SPF: Specific-pathogen-free
IL-1β: Interleukin-1 beta
IL-6: Interleukin-6
TNF-α: Tumor necrosis factor-alpha
MCP-1: Monocyte chemoattractant protein-1
MIP-2: Macrophage inflammatory protein-2
IRAK1: Interleukin 1 receptor-associated kinase 1
TRAF6: TNF receptor-associated factor 6
TAB2: TGF-beta-activated kinase 1 binding protein 2
EGFR: Epidermal growth factor receptor
NF-κB: Nuclear factor kappa B
MAPK: Mitogen-activated protein kinase
JNK: c-Jun N-terminal kinase
ERK: Extracellular regulated protein kinases
GAPDH: Glyceraldehyde-3-phosphate dehydrogenase
UTR: Untranslated region
